# Impact of tapping and soil water status on fine root dynamics in a rubber tree plantation in Thailand

**DOI:** 10.3389/fpls.2013.00538

**Published:** 2013-12-25

**Authors:** Naruenat Chairungsee, Frederic Gay, Philippe Thaler, Poonpipope Kasemsap, Sornprach Thanisawanyangkura, Arak Chantuma, Christophe Jourdan

**Affiliations:** ^1^Office of Agricultural Research and Development Region 1Chiang Mai, Thailand; ^2^CIRAD, UMR Eco&SolsMontpellier, France; ^3^Hevea Research Platform in Partnership, DORAS Centre, Kasetsart UniversityBangkok, Thailand; ^4^Faculty of Agriculture, Kasetsart UniversityBangkok, Thailand; ^5^Faculty of Science, Kasetsart UniversityBangkok, Thailand; ^6^Chachoengsao Rubber Research Center, Department of AgricultureChachoengsao, Thailand

**Keywords:** *Hevea brasiliensis*, fine root dynamics, root elongation rate, fine root production, soil water content, field rhizotrons, seasonal climatic variations, Thailand

## Abstract

Fine roots (FR) play a major role in the water and nutrient uptake of plants and contribute significantly to the carbon and nutrient cycles of ecosystems through their annual production and turnover. FR growth dynamics were studied to understand the endogenous and exogenous factors driving these processes in a 14-year-old plantation of rubber trees located in eastern Thailand. FR dynamics were observed using field rhizotrons from October 2007 to October 2009. This period covered two complete dry seasons (November to March) and two complete rainy seasons (April to October), allowing us to study the effect of rainfall seasonality on FR dynamics. Rainfall and its distribution during the two successive years showed strong differences with 1500 and 950 mm in 2008 and 2009, respectively. FR production (FRP) completely stopped during the dry seasons and resumed quickly after the first rains. During the rainy seasons, FRP and the daily root elongation rate (RER) were highly variable and exhibited strong annual variations with a total FRP of 139.8 and 40.4 mm^-^^2^ and an average RER of 0.16 and 0.12 cm day^-^^1^ in 2008 and 2009, respectively. The significant positive correlations found between FRP, RER, the appearance of new roots, and rainfall at monthly intervals revealed the impact of rainfall seasonality on FR dynamics. However, the rainfall patterns failed to explain the weekly variations of FR dynamics observed particularly during the rainy seasons. At this time step, FRP, RER, and the appearance of new FR were negatively correlated to the average soil matric potential measured at a depth of between 30 and 60 cm. In addition, our study revealed a significant negative correlation between FR dynamics and the monthly production of dry rubber. Consequently, latex harvesting might disturb carbon dynamics in the whole tree, far beyond the trunk where the tapping was performed. These results exhibit the impact of climatic conditions and tapping system in the carbon budget of rubber plantations.

## INTRODUCTION

Changes in terrestrial carbon stocks have significantly contributed to the increase of greenhouse gases (GHGs) in the atmosphere (Houghton, 1999). Land use changes (deforestation–afforestation) are important drivers of the global carbon balance. Forest conversion can have a profound effect on the carbon cycle (Lal, 2005; Jandl et al., 2007; Li et al., 2008) and large areas of the remaining tropical rainforests are being logged and converted to agricultural systems at high rates (Nepstad et al., 1999; Achard et al., 2002). In the tropical belt, and more particularly in southeast Asia, the rapid expansion of tree plantations (mainly oil palm, rubber, and coffee) has been among the main causes of deforestation in the last 20 years (Ziegler et al., 2012; Chiti et al., 2013; Ramdani and Hino, 2013). Conversely, tree plantations have expanded also onto degraded or marginal lands where they could contribute to the rehabilitation of those lands (Sang et al., 2013). A benefit of tree plantations is that in addition to timber and agricultural products (such as fruits and latex), they are forest-like ecosystems that can improve some ecosystem services like water regulation, soil fertility, and carbon sequestration in the soil (Vihervaara et al., 2012). However, the appropriate plantation management should be applied to optimize those ecosystem services.

Due to the increasing world demand for natural rubber, most of the countries producing natural rubber (*Hevea brasiliensis*) have supported the expansion of rubber plantations in “non-traditional” environments particularly in Thailand and China (Fox and Castella, 2013). In southern China, rubber plantations have been set up at the expense of secondary forests causing a significant loss in the soil carbon stock (de Blécourt et al., 2013). In Thailand, the top producer of natural rubber in the world, rubber plantations have expanded to the north-eastern part of the country where they have replaced mainly cash crops like sugar cane. In these new planting areas, the sustainability of rubber plantations is challenged by sub-optimal weather conditions (drought, low temperature), the low fertility of most of the soils (Boithias et al., 2011; Isarangkool Na Ayutthaya et al., 2011; Clermont-Dauphin et al., 2013), and the variability of the typical monsoon climate prevailing in mainland southeast Asia (Bridhikitti, 2013). Little is known about the carbon balance of rubber plantations under these particular conditions or about the best management practices for the optimum carbon sequestration in the soil. Wauters et al. (2008) found a 46% decrease in the carbon stock of the standing biomass of rubber plantations grown in sub-optimal conditions in Brazil compared to plantations in Ghana. Satakhun et al. (2013) showed that the soil water content (SWC) is the main driver of soil respiration in a rubber plantation under a sub-optimal rainfall regime with higher rates of soil CO_2_ efflux during the rainy season and lower rates during the dry season. The dynamics of fine roots (FRs) in a rubber plantation have not been studied in details yet despite the fact that the belowground C allocation is a major component of the carbon balance, depending largely on FR production (FRP), mortality and turnover (Jackson et al., 1997; Matamala et al., 2003). In addition, FRs play an essential role in the acquisition of water and essential nutrients, while at the ecosystem level, they make a significant contribution to biogeochemical cycling (Pregitzer et al., 2002). A better understanding of FR dynamics is therefore important in the design of an appropriate management plan for the plantations (timing of fertilization, control of understorey, etc.). Previous studies on the root system of rubber trees were mainly conducted on seedlings either in field or in greenhouse conditions (Le Roux and Pagès, 1994; Thaler and Pagès, 1996a, b). To our knowledge, only two papers reported studies about root dynamics in mature plantations. George et al. (2008) determined the active root distribution pattern of rubber trees by the radioassay of latex serum which revealed that 55% of the root activity was confined to the top 10 cm of the soil layer and that root activity declined with increasing soil depth and the concentration of physiologically active roots at 90 cm depth was only 6%. Gonkhamdee et al. (2009) used a permanent access well 4.5 m deep equipped with rhizotrons to monitor FR appearance/disappearance during 17 months in a rubber plantation in Thailand. They showed how FR dynamics changed with time at different depths but their study did not provide any quantitative analysis of the relationships between FR dynamics and the environmental conditions or the stand characteristics.

A number of studies have demonstrated that FR growth was influenced by both exogenous and endogenous parameters (Moroni et al., 2003; Tierney et al., 2003). For exogenous factors, Konôpka et al. (2007) reported that drought stress was most intense for FRs in the topsoil and weakest for FRs in the deepest soil layers. Studies of forest in which the rainfall is highly seasonal have shown that the roots grow mostly in the rainy season (Kavanagh and Kellman, 1992; Lopez et al., 1998) and die during the dry season (Srivastava et al., 1986; Kummerow et al., 1990). Although these patterns suggest direct control by soil water availability, growth also coincides with the leaf flush in the canopy and a very sharp increase in the soil nutrient availability as the rains begin (Singh et al., 1989; Roy and Singh, 1995). The endogenous factors controlling the root growth are mainly linked to the development of the aerial part and the partitioning of carbon resources between the aboveground and belowground parts of the tree. Research by Thaler and Pagès (1996a, b) on rubber tree seedlings showed that both the apical diameter and the elongation rate of roots were depressed during the period of shoot growth. This may be related to carbon availability, as Singh and Srivastava (1986) found that the total non-structural carbohydrate content of teak (*Tectona grandis*) FRs was highest during the dry summer and lowest in the early part of the rainy season. In this regard, the carbon dynamics in the rubber tree present two specific features. First, the trees completely shed their leaves and produce new leaves every year over a period of 4–5 weeks, called wintering (Sabu and Vinod, 2009). In the marginal areas of Thailand, this wintering period happens in the middle of the dry season when the SWC, leaf gas exchange, and radial growth are at their lowest (Carr, 2011). Secondly, the carbon allocation in the trunk is strongly modified when the tree is tapped to harvest latex; radial growth is depressed (Silpi et al., 2006) and non-structural carbohydrates are diverted to build up trunk reserves (Silpi et al., 2007). Several methods have been used to estimate the FR biomass, production, and turnover (Santantonio and Grace, 1987; Hendrick and Pregitzer, 1992; Nadelhoffer and Raich, 1992). The sequential soil core method has been used widely (Persson, 1980, 1983; Ahlström et al., 1988; Comeau and Kimmins, 1989; Yin et al., 1989), but this method provides only a momentary representation of the FR biomass; the actual growth of FRs cannot be followed (Makkonen and Helmisaari, 1999). Direct observation methods of root dynamics are now commonly applied on a field scale using transparent acrylic tubes (mini-rhizotrons) or transparent panes of glass (rhizotrons) inserted in the soil (Jourdan and Rey, 1997; Hendricks et al., 2006; Jourdan et al., 2008). They allow the direct measurement of the appearance, disappearance, speed of growth, mortality, and lifespan of individual roots (Keyes and Grier, 1981) at a high temporal frequency (Metcalfe et al., 2007). Rhizotrons have several advantages over most of the other root study methods (Taylor et al., 1990; Box, 1996) as they allow the determination of the seasonal pattern of root growth and periods of minimal and maximal root growth (Vogt et al., 1998). Such non-destructive techniques are also important when dynamic changes of the roots in response to the environment are to be studied (McMichael et al., 1992). The disadvantages of rhizotrons are: the difficulty of precisely measuring very small roots, especially in the upper few millimeters of the soil (Vos and Groenwold, 1987); and the root growth disturbance effects of the window installation (Joslin and Wolfe, 1999; Coleman et al., 2000). However, no technique has been accepted universally as the best (Jourdan et al., 2008).

The current study presents the dynamics of FRs observed with flat rhizotrons during two successive years in a mature rubber plantation grown in a non-traditional area of Thailand, with sub-optimal annual rainfall for rubber cultivation (i.e., below 1500 mm), a 4- to 5-month dry season and intermittent spells of drought during the rainy seasons. This study was conducted in the framework of the Rubberflux project which aims to quantify the carbon, water, and energy budget of a rubber plantation (Thaler and Kasemsap, 2007). In this regard, this study had two main objectives. The first objective was to study the relations between FR dynamics and other components of the net primary productivity (NPP) of the stand such as leaf phenology, stand growth, and latex harvesting. According to previous soil respiration studies conducted on the same site (Satakhun et al., 2013), we assumed that the FR growth would be lower in the dry season than in the rainy season as suggested by the soil CO_2_ efflux dynamics. The second objective was to assess the impact of climatic factors on FR dynamics, particularly the inter- and intra-annual variability of the rainfall regimes.

## MATERIALS AND METHODS

### SITE DESCRIPTION

The experimental site was located at the Chachoengsao Rubber Research Center, Chachoengsao province (13°41′N, 101°04′E, and 69 m elevation), eastern Thailand. The observation plot was a monoclonal stand of rubber trees (*Hevea brasiliensis* Müll. Arg.) planted with the clone RRIM 600 in 1994 after cassava cultivation, with a tree spacing of 7 m × 2.5 m. The clone RRIM 600 is the most extensively planted in Thailand (78% of the planted area). Tapping for latex production began when the trees were 9 years old in 2003. Since then, the trees have been tapped each year during the 9 months from late April/early May to the end of January. During this period, tapping was performed every two or three days with a half-spiral downward cut [(1/2) S d/2, (1/2) S d/3]. The average diameter of the trees at 1.70 m from the ground was 20.04 cm (3.95 cm standard deviation) at the beginning of the study in November 2007.

The soils in the plot belong to the Kabin Buri series with 50% sand, 15% silt, and 35% clay. The soil depth is limited at 1–1.5 m by a compact layer of ferralitic concretions that strongly limits root growth. The mean annual air temperature and cumulative rainfall were 28.1°C and 1328 mm, respectively, with a strict dry season between November and April (sourced from the Thai Meteorological Department).

### MONITORING OF FINE ROOT DYNAMICS

Fine root dynamics were monitored from November 2007 to October 2009 using flat rhizotrons (Jourdan and Rey, 1997) installed in the vicinity of three trees. The selected trees had a girth at breast height in the range of the average girth of the plot and had no dead trees in their immediate surroundings. Two types of rhizotron were installed for each tree at a distance of about 1.5 m from the base of the trunk in the inter-row: one near-horizontal rhizotron with an inclination of 20° from the horizontal and one near-vertical rhizotron with an inclination of 20° from the vertical. Each rhizotron was made of a square-shaped piece of Plexiglas pane (0.8 m × 0.8 m) reinforced with a metal frame. The depth of soil explored by the rhizotrons was 27 cm for the near-horizontal one and 75 cm for the near-vertical one to characterize the shallow- and “deep”-FR dynamics, respectively.

The six rhizotrons were set up in September 2007 on the soil wall toward the tree in trenches 1 m wide by 1 m long and 30 cm deep for the near-horizontal and 100 cm deep for the near-vertical units. A 2–3 cm layer of 2-mm sieved soil prepared when the trenches were dug was inserted between the soil wall of the trench and the Plexiglas pane and compacted as much as possible to reach the former soil compaction and to provide a good contact between the transparent pane of glass and the sieved soil. Each transparent screen was covered by double-layer black plastic sheets to prevent light from hindering the root growth. The trenches were covered by a metal roof to protect the rhizotrons from direct sunlight, rainfall, insects, and rodents.

Observation of the appearance, growth, and development of the roots started 3 weeks after the installation of the rhizotrons. Every week from November 6, 2007 to October 19, 2009, we traced the new segments of roots, linked to the growth of the existing roots or the apparition of a new root in the rhizotron, using permanent colored markers on a transparent plastic sheet fixed on the Plexiglas of each screen. A different color marker was used on each sampling date.

Every transparent plastic sheet filled with root drawings was digitized manually using a 61 cm × 91 cm format digitizer (Summagrid V, GTCO CalComp Inc., Columbia, MD, USA) and the RhizoDigit software (CIRAD, Montpellier, France). The RhizoDigit software facilitated the generation and management of the database including the date of apparition of each root segment and its length at each observation date and for each root diameter class.

### ENVIRONMENTAL CONDITIONS OF THE STUDY

Daily data of the average air temperature, cumulative rainfall, and cumulative photosynthetic active radiation (PAR) were computed from 30-min data continuously measured at the top of a 25-m-high tower set up in the center of the observation plot (Thaler and Kasemsap, 2007). Every month during the observation period, soil samples at 20, 40, and 60 cm were collected in three locations near each rhizotron; the samples were used to determine the water content after oven drying for 24 h at 105°C. In June 2008, manual tensiometers (Raindrop, Eastern Agritek Co., Rayong, Thailand) were installed at soil depths of 30 and 60 cm at three locations close to each selected tree, that is, near each pair of horizontal and vertical rhizotrons. The soil matric potential (SMP) was recorded once every 2 days from July 3, 2008 to October 21, 2009 except between January 1, 2009 and June 14, 2009 because the soil was too dry during this period to measure the SMP with the tensiometers.

### STAND CHARACTERISTICS: PAI, GIRTH INCREMENT, LEAF LITTER, AND LATEX YIELD

The plant area index (PAI; i.e., leaf plus branch area index) of the stand was measured from hemispherical pictures of the canopy taken in the vicinity of the rhizotrons with a Nikon Coolpix 995 camera and a Nikon FC-E8 fish-eye lens. All pictures were analyzed using the GLA software (Institute of Ecosystem Studies, Simon Fraser University, Burnaby, Canada).

Leaf litter samples were collected every 2 weeks in twenty 1-m^2^ litter traps randomly positioned in the stand. The dry biomass of the litter was measured after drying the samples at 60°C until constant weight. The girth of the trees at 1.7 m above the ground was measured once a year at the time of leaf shedding. The latex yield was determined monthly by weighing the rubber coagulum. The total solid content was measured on a sub-sample in order to convert the fresh weight into grams of dry rubber. Those measurements were used to calculate the components of the aboveground NPP of the plantation, namely, the annual increment of tree girth as a proxy of the annual increment in standing biomass, the annual latex production and litter biomass (t ha^-^^1^). We calculated those variables for the physiological cycle of the rubber trees, i.e., the period between the annual wintering period marked by the complete shedding of the leaves and the quick regrowth that follows (between January 23, 2008 and January 31, 2009 and between February 4, 2009 and January 16, 2010).

### DATA ANALYSIS

The root elongation rate (RER, cm day^-^^1^) was computed from the database generated by the RhizoDigit software as shown in Eq. 1:

(1) $$RER=\left(RL_{d2}-RL_{d1}\right)/\left(d_{2}-d_{1}\right)$$

where RL_d1_ and RL_d2_ are the length (cm) of a root segment between the two dates of observation (d_1_ and d_2_). In addition, the RhizoDigit database was used to count the total number of roots in each rhizotron and among all these roots, the number of new roots, the number of growing roots, and the number of paused roots (Thaler and Pagès, 1996a, b; Nodichao et al., 2011) for each observation date. Because the mortality of roots is difficult to estimate through a transparent screen, we have defined “paused roots” as the roots that exhibited no elongation in length and diameter between two or more successive observation dates. The paused roots turned into the “dead roots” category when the absence of growth was persistent over 2 months along with morphological and color changes. The FRP (mm^-^^2^) on a seasonal or annual pattern was assessed by summing the total root length produced between two successive dates in the corresponding period divided by the related observation screen area (m^2^) of the rhizotron. Next, the average value at each date of observation (i.e., every week) of the RER, FRP, total number of roots, and number of roots of different categories (new, growing, paused), was computed for the six rhizotrons. The monthly average RER and FRP, or the sum of root numbers, were also calculated when the SWC was measured. These weekly or monthly data were analyzed against climatic, soil water status, and PAI data using an LSD test for comparison of mean values, the Pearson multiple correlation test, and non-linear regression performed with the Xlstat software (Addinsoft, Paris, France).

## RESULTS

### CLIMATIC CONDITIONS DURING THE MEASUREMENT PERIOD

**FIGURE 1:**
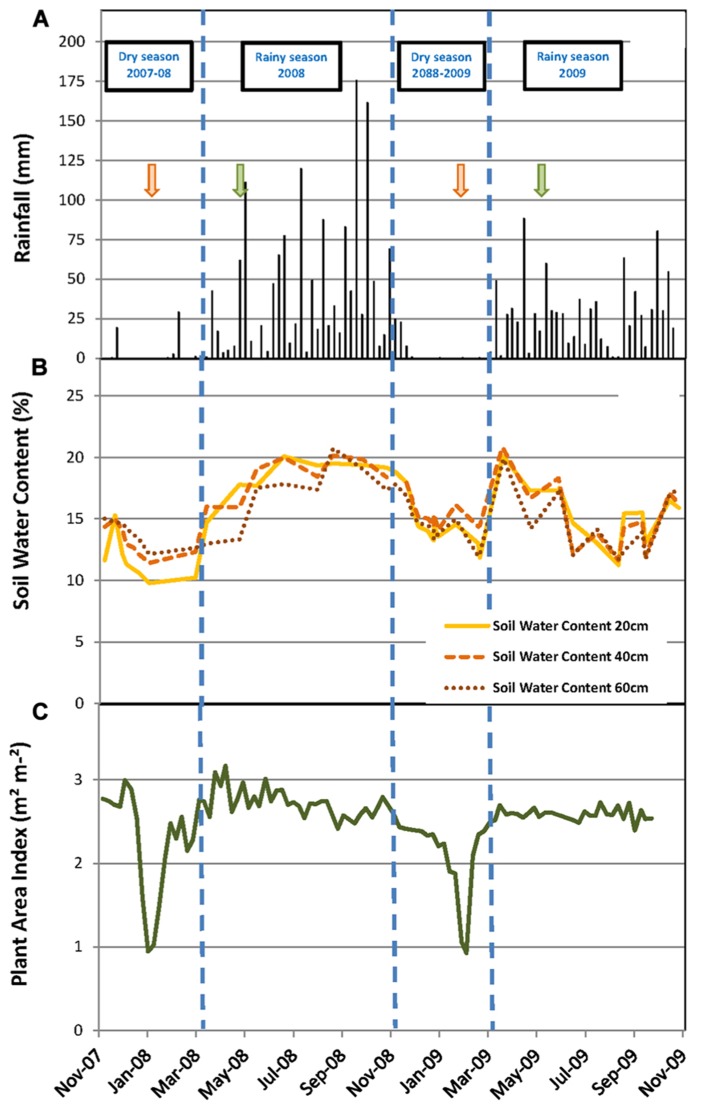
**Environmental and stand conditions during the experiment.** **(A)** Weekly cumulative rainfall (mm); soil water content (%) measured every month at soil depths of 20, 40, and 60 cm; **(C)** plant area index (m^2^ m^-^^2^) measured weekly. Vertical dashed lines indicate the limits of dry and rainy seasons. Green and orange arrows mark the beginning and the end of the tapping season, respectively.

The daily rainfall pattern during the measurement period showed the succession of rainy and dry seasons (**Figure 1A**). The dry seasons extended from early November to mid-March in both years. These dry seasons were characterized by only five rainy days (days with more than 1 mm of rain), and a total cumulative rainfall of 53 mm for the dry season in 2007–2008 and 44 mm for the dry season in 2008–2009 (**Table 1**). The rainy seasons, though extending over the same months in both years, showed contrasting figures; the cumulative rainfall and the number of rainy days were 1500 mm and 88 days in 2008 and 952 mm and 76 days in 2009, respectively. The daily mean air temperature and cumulative PAR did not show marked seasonal trends. The temperature varied between 20 and 30°C, and PAR varied between 13.8 and 50.1 mol m^-^^2^ day^-^^1^.

**Table 1:** Statistics for variables describing the environmental conditions of the study and fine root dynamics for each of the four seasons identified based on the rainfall regime during the observation period.

	Dry season (November 2007 to March 2008)	Rainy season (April to October 2008)	Dry season (November 2008 to March 2009)	Rainy season (April to October 2009)
Cumulative rainfall (mm)	53.0	1499.8	43.8	952.3
Rainy days (day)	5	88	5	76
Air temperature (^°^C)	25.7 (b)	26.1 (ab)	24.8 (c)	26.2 (a)
PAR (mol m^-^^2^ day ^-^^1^)	34.6 (ab)	35.2 (ab)	33.8 (b)	36.1 (a)
Soil water content (%)	13.0 (c)	18.5 (a)	15.0 (b)	15.7 (b)
Soil matric potential (MPa)	NA	0.0137 (a)	NA	0.0395 (b)
PAI (m^2^ m^-^^2^)	2.3 (b)	2.7 (a)	2.2 (b)	2.6 (a)
Fine root production (cm m^-^^2^ week ^-^^1^)	122.1 (b)	395.8 (a)	34.8 (c)	116.2 (b)
Root elongation rate (cm day ^-^^1^)	0.08 (bc)	0.16 (a)	0.04 (c)	0.12 (b)
Number of roots (week ^-^^1^)	119.8 (b)	168.6 (a)	36.2 (d)	67.4 (c)
Number of growing roots (week ^-^^1^)	23.7 (b)	57.5 (a)	6.3 (c)	22.7 (b)
Number paused roots (week ^-^^1^)	74.1 (a)	52.8 (b)	23.2 (c)	24.7 (c)
Number of new roots (week ^-^^1^)	16.9 (bc)	51.0 (a)	5.1 (c)	19.9 (b)
% of growing roots (week ^-^^1^)	19 (b)	31 (a)	13 (b)	33 (a)
% of paused roots (week ^-^^1^)	63 (a)	36 (b)	75 (a)	38 (b)
% of new roots (week ^-^^1^)	13 (b)	28 (a)	9 (b)	29 (a)

*Each number is the seasonal average of weekly or daily data except for cumulative rainfall and rainy days which are sums. Numbers with different letters in parentheses on the same line are significantly different at *p* < 0.05 (LSD test). NA, data not available.*

### SOIL WATER STATUS

The SWC at 20, 40, and 60 cm soil depth varied from 9.8 to 20.1% during the measurement period (**Figure 1B**). The highest values of the SWC were reached during the rainy seasons and the lowest during the dry seasons. However, the dynamics of the SWC were different in the two years, particularly during the rainy season. In 2008, the SWC increased progressively from 12% in February to 18% in May and then varied a little between 18 and 20% until the end of the rainy season in October. Conversely in 2009, the SWC increased rapidly from 13% in February to 20% in March, then decreased to 11.5% in August and varied between 11 and 17% until the end of the rainy season (**Figure 1B**). Consequently, the average SWC during the rainy season was significantly higher in 2008 than in 2009 (18.5 versus 15.7%; **Table 1**). The same results were observed for the average SMP measured at 30 and 60 cm soil depth during the rainy season (**Table 1**).

### STAND CHARACTERISTICS

The PAI varied between 1.0 and 3.2 m^2^ m^-^^2^ (**Figure 1C**). The PAI values were significantly higher during the rainy seasons compared to the dry seasons (**Table 1**). In both years, the PAI dropped sharply to a value of 1 m^2^ m^-^^2^ during the dry season and increased to 2.5 m^2^ m^-^^2^ within the following 3–4 weeks (**Figure 1C**). These data illustrate the wintering period of the rubber trees with complete leaf shedding followed by quick leaf regrowth that occurs annually in clone RRIM 600 rubber tree plantations. The increase in the average girth of the trees at 1.7 m over the physiological cycle of the trees was +2.1% in 2008 and +2.5% in 2009. Similarly, dry rubber production was higher in 2009 (1.38 t ha^-^^1^) than in 2008 (1.16 t ha^-^^1^) while the aboveground litter production was lower in 2009 (1.21 t ha^-^^1^) than in 2008 (1.31 t ha^-^^1^).

### FINE ROOT DYNAMICS

The root dynamics observed through horizontal and vertical rhizotrons did not exhibit any significant differences either when compared to the same soil horizon prospected by roots nor to different soil depths. Consequently, results presented hereafter were issued from both horizontal and vertical rhizotrons.

**FIGURE 2:**
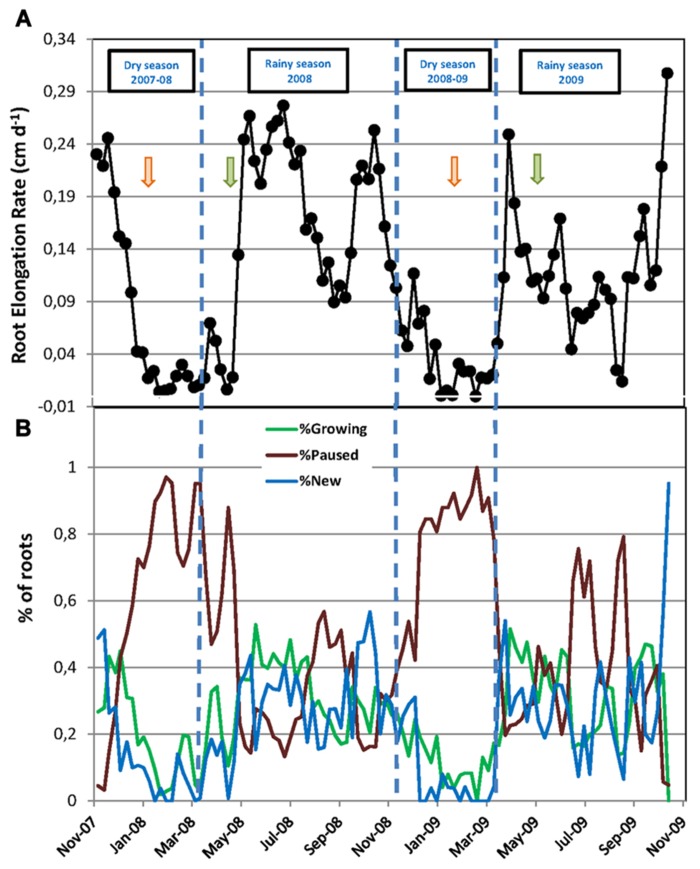
**Fine root dynamics of mature rubber trees during two successive years.** **(A)** Root elongation rate (cm day^-^^1^) calculated between two dates of observation as the increase in root length during the interval over the number of days between the two measurements; **(B)** Percentage of new roots, growing roots, and paused roots relative to the total number of roots in the rhizotron at each date of measurement. In **(A,B)**, every data point is the mean of six rhizotron measurements. Vertical dashed lines indicate the limits of dry and rainy seasons. Green and orange arrows mark the beginning and the end of the tapping season, respectively.

The total number of roots and the numbers of new, growing, and pause roots were significantly higher in the first year of the experiment, from November 2007 to October 2008, compared to the second year, from November 2008 to October 2009 (**Table 1**). Consequently, we calculated the numbers of new, growing, and paused roots as a percentage of the total root number at each observation in order to account for these differences when comparing the root dynamics between the two years. The data transformed this way showed a remarkably similar pattern over the two years, with a sharp decrease in the number of new and growing roots at the beginning of the dry seasons along with a sharp increase in the number of paused roots up to 100% (**Figure 2B**). Root growth and production resumed when the rainy seasons started and then varied between 0 and 52–56%. The average percentage of each category of roots was not significantly different between the two rainy seasons and the two dry seasons (**Table 1**).

The RER and FRP were also strongly affected by the alternation of the dry and rainy seasons (**Figure 2A**; **Table 1**). In both dry seasons, the RER decreased quickly from values above 0.25 cm day^-^^1^ in early November to less than 0.05 cm day^-^^1^ at the end of December in each observed year. Then, the RER remained below 0.05 cm day^-^^1^ until the onset of the rainy season at the end of March. FRP in the same dry periods remained low with 27.1 and 8.0 m of cumulated FR length per m^-^^2^ of observation screen area for 2008 and 2009, respectively (**Table 1**). During the rainy seasons, the RER varied from 0.01 to 0.31 cm day^-^^1^ (**Figure 2A**) with an average of 0.16 and 0.12 cm day^-^^1^ in 2008 and 2009, respectively (**Table 1**). The average RER and FRP values during the rainy season were significantly higher in 2008 than in 2009 (**Table 1**). This resulted in a total FRP of 139.8 m m^-^^2^ over the tree physiological cycle from the end on January 2008 to the end of January 2009. FRP was not measured over the whole cycle from January 2009 to January 2010. However, data from 2008 showed that FRP between October 2008 and January 2009 accounted for only 4% of FRP in the 2008–2009 cycle. The total FRP for the 2009–2010 cycle could thereby be estimated as 40.4 m m^-^^2^, that is, 71% lower than in 2008–2009.

### CORRELATION BETWEEN FINE ROOT DYNAMICS, ENVIRONMENTAL CONDITIONS, AND STAND CHARACTERISTICS

Table 2 shows the Pearson coefficients of correlation between the variables describing the environmental conditions during the study, PAI and the variables related to the FR dynamics, namely the RER, FRP, and the percentage of growing roots, paused roots, and new roots. The test was performed first on the data computed with a monthly time step over the entire study period in order to include the data on the SWC. Using this time step, we found a significant positive correlation between FR dynamics and rainfall, the number of rainy days and the PAI. On the other hand, we found a significant negative correlation between FR dynamics and dry rubber production.

**Table 2:** Correlation coefficients (Pearson test) between variables describing the environmental conditions of the study, the stand characteristics and the variables related to the fine root dynamics.

	Root elongation rate (cm day^-^^**1**^)	%Growing roots	% Paused roots	% New roots	Fine root production (cm m^-^^**2**^ week ^-^^**1**^)
**Monthly data (all seasons)**
Air temperature	-0.070	0.148	0.002	-0.053	0.083
Rainfall	0.605**	0.457*	-0.591**	0.656**	0.566**
Rainy days	0.557**	0.539**	-0.617**	0.668**	0.468*
PAR	0.108	0.282	-0.147	0.067	0.115
Plant area index	0.545**	0.551**	-0.584**	0.550**	0.459*
Dry rubber production	-0.582*	-0.618**	0.591*	-0.582*	-0.678**
Soil water content	0.439*	0.270	-0.370	0.391	0.456*
Soil matric potential	NA	NA	NA	NA	NA
**Weekly data (rainy seasons only)**
Air temperature	-0.309*	0.045	0.319**	-0.179	0.022
Rainfall	0.115	-0.010	-0.084	0.068	0.052
Rainy days	0.100	0.044	-0.115	-0.001	-0.053
PAR	0.001	0.160	-0.047	0.096	0.125
Plant area index	0.138	0.103	-0.107	0.022	0.337
Dry rubber production	NA	NA	NA	NA	NA
Soil water content	NA	NA	NA	NA	NA
Soil matric potential	-0.770***	-0.154	0.627***	-0.383*	-0.548***

Upper half of the table shows the correlation between data computed monthly for both years of the study. Lower half of the table shows the correlation between the data computed weekly during the two rainy seasons only. The soil water content and soil matric potential data used for these tests were the average of the measurements done at three and two different depths, respectively. Bold numbers indicate significant correlations at *p < 0.05, **p < 0.01, and ***p < 0.001. NA, data not available.

**FIGURE 3:**
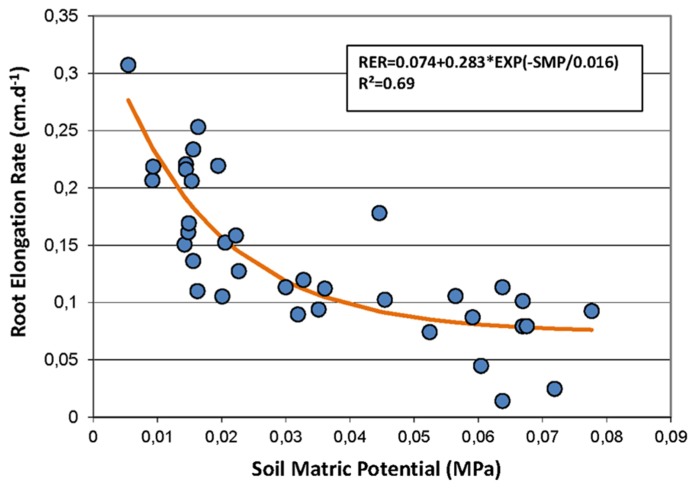
**Non-linear regression between the soil matric potential (SMP, MPa) and the root elongation rate (RER, cm day^**-**^^**1**^).** Symbols represent the measured data, and the line shows the non-linear model fitting the data. The model is RER = 0.074 + 0.283 × exp(-SMP/0.016).

Secondly, the Pearson correlation test was performed on weekly data during the rainy seasons only to study the short-term dynamics of FRs with information on the short-term dynamics of the soil water status provided by the measurement of the SMP. Using this time step, the RER was not correlated with rainfall but was negatively correlated with the air temperature and strongly negatively correlated with the SMP. **Figure 3** shows that the relationship between the RER and the SMP is well fitted by a negative exponential model. The percentages of the FRP and the new and paused roots were also correlated to the SMP. The percentage of growing roots did not show any correlation with the measured environmental conditions.

## DISCUSSION

This study is the first detailed assessment of FR dynamics (the RER, FRP, and FR status) in a rubber tree plantation. There was a strong decrease in every measured parameter (number of the different types of roots, FR elongation rate, and FRP) between the first (2008) and the second (2009) year of the experiment. This may have been due to the rhizotron methodology. Disturbance of roots and of the rooting environment during the rhizotron installation may have been offset by an overproduction of roots during the weeks or months after the installation (de Ruijter et al., 1996; Vogt et al., 1998). Consequently, it is generally recommended to wait a certain period after the installation of a rhizotron before starting any measurement of FR dynamics along the glass surface. In some species, this lag time could be up to 3–8 months according to the stabilization of the FR standing length (Green et al., 2005; Hendricks et al., 2006; Metcalfe et al., 2007). Other potential sources of error with rhizotron approaches may be the effect of the observation window on root longevity (Withington et al., 2003) and the difficulty to distinguish the senescent process of FRs leading to many biases in the estimation of the amount of dead roots (Stevens et al., 2002) and globally the mortality process. Wang et al. (2005) also showed that a nutrient depletion zone at the root–rhizotron interface could be observed after several months and could lead to a decrease in the occurrence of new roots in the rhizotron (Mao et al., 2013). It is difficult to say if the growth of FRs was affected by any offset growth or the depletion of nutrient at the soil–rhizotron interface in our study. Nevertheless, our results clearly showed that the development pattern of the FRs was remarkably similar for the two years. Therefore, we can conclude that despite a possible impact on the number of roots, the rhizotrons used in our study provided reliable data on the dynamics of the FRs of rubber trees.

The average RER of the rubber trees in the 2008 wet season was 0.16 cm day^-^^1^ and only 0.12 cm day^-^^1^ in October 2009, with a maximum value of 0.30 cm day^-^^1^ in both years. These rates are lower than for common tree roots (0.3–0.5 cm day^-^^1^; Kramer and Boyer, 1995) and lower than for other tropical trees such as eucalypts (from 0.6 to 1.5 cm day^-^^1^; Misra, 1999; Thongo M’Bou et al., 2008) or oil palm grown in the Côte d’Ivoire (0.3 cm day^-^^1^; Jourdan and Rey, 1997). The lower rate of root elongation in the rubber trees in the current study might have been due to the depressing effect on tree growth of the tapping for latex production as was shown by the negative correlation found between FR dynamics and the dry rubber yield using the monthly time step. The negative impact of tapping (i.e., severing a thin slice of bark on a regular basis to collect the latex contained in the laticifer vessels of the phloem) on the aboveground rubber tree biomass, growth, and carbohydrates allocation at the trunk scale has been well studied for decades (from Pyke, 1941 to Silpi et al., 2007). The results of Silpi et al. (2006) showed a sharp decline in the radial growth of tapped trees compared to untapped trees within 2 weeks from the beginning of the tapping season. It illustrates the strength of the carbon sink created by tapping and the competition between this new sink and the primary growth. More surprisingly, Silpi et al. (2007) showed that tapping increased the storage of carbohydrates as reserves in the trunk, thereby increasing the strength of this sink and the overall competition for carbohydrates at the trunk level. Besides competition for carbon resources, tapping may also result in a limitation of carbohydrates transportation below the tapping cut due to the disruption of the phloem tissues on this part of the trunk (Silpi et al., 2007). FR dynamics are likely to be affected by these important changes in the carbon dynamics at the trunk level. Both the FRP and life span are indeed very sensitive to changes in the sink strength of the aboveground parts of trees, either due to the phenology of the shoots and leaves or due to the management of the trees for example by pruning (Comas et al., 2000; Pregitzer, 2003; Steinaker and Wilson, 2008). In rubber trees, Thaler and Pagès (1996b) showed that root growth was depressed every time a new flush of leaves was produced. Interestingly, they found that the number of paused roots increased during periods of leaf growth. We also found a positive correlation between the percentage of paused roots and dry rubber production. Comparing the FR dynamics of the tapped and untapped trees would be relevant to confirm the impact of tapping on the root system and to investigate the underlying mechanisms. In this regard, it would be interesting to test several tapping systems corresponding to a gradient in tapping intensity (Lacote et al., 2010), and thereby to a gradient in the strength of the latex sink to establish a response curve between FR and latex production.

Root growth is not only influenced by endogenous factors linked to carbohydrates availability but also by exogenous factors related to environmental conditions (Moroni et al., 2003; Tierney et al., 2003). In our study, rainfall and the soil water status clearly appeared as the main environmental drivers of FR dynamics, whereas other climatic factors had less effect. This is consistent with previous works on tree plantations (Thongo M’Bou et al., 2008), or forest stands in tropical conditions (Green et al., 2005). First, we observed a similar seasonal trend in FR growth and development during the two different years which is consistent with the succession of the dry and rainy seasons. This observation was confirmed by the good correlations between the root parameters and the rainfall characteristics using a monthly time step. Root growth almost stopped during the dry season and quickly resumed at the onset of the rainy season. This was linked to the proportion of growing roots and the production of new roots, in a similar manner to the results from FR dynamics in a tropical forest (Green et al., 2005). However, it is noteworthy that during the dry season, a large proportion of roots (up to 100%) stopped growing but did not die, as they resumed growing in the next rainy season. Secondly, we also observed significant differences in root growth between the two rainy seasons, with a 25% reduction in the average RER of the 2009 rainy season compared to the 2008 rainy season. The 2009 rainy season was remarkably dryer than in 2008 with 36% less rainfall (952 mm in 2009 versus 1500 mm in 2008), resulting in a 3% reduction in the average SWC. These results are consistent with those of Meier and Leuschner (2008) who found a 30% decrease in the FR biomass when the rainfall was reduced by 40%. However, the weekly variations in the RER during the rainy seasons, characterized particularly by a sharp decrease in the RER in August of both years, were more surprising. These variations could be explained neither by the rainfall events used with this time step nor by the evolution of the SWC, which remained rather high during all of the second half of the 2008 rainy season. A closer assessment showed that there was a clear negative relationship between the SMP and the FR elongation rate. This showed that FR growth was closely dependent on the soil water availability, in ranges of the SMP (below -0.05 MPa) that would hardly result in measurable changes in the soil volumetric content. To our knowledge, such a relationship between the SMP and the FR growth of field-grown trees has not been shown before. Previously, Kuhns et al. (1985) using black walnut trees and Bengough et al. (2011) using several annual crops showed a sharp decrease of FR growth at much lower SMPs (between -0.5 and -1.0 MPa). The values of the SMP reported in these papers were taken at or close to the root surface. In our study, it is likely that the SMP at the surface of the roots growing in the rhizotron was lower than the readings of the tensiometers installed a few meters away from the rhizotrons. However, this relationship suggests that the FR growth of rubber trees was very sensitive to water stress in this study as was shown previously by Chiatante et al. (1999) using pine saplings and by Konôpka et al. (2007) using Japanese cedar.

These contrasted conditions for rainfall and the soil water status between the two years resulted in a reduction of 71% in the total FRP in the rhizotrons in 2009 compared to 2008. The other components of the NPP did not show such a big variation. The aboveground litter production was only reduced by 8% in 2009 compared to 2008 while the girth increment and latex production were higher in 2009 than in 2008. This would suggest that the aboveground parts of the trees were less sensitive than the FRs to the seasonal drought and the water stress events during the rainy seasons. Those differences in the sensitivity of NPP parameters to a variation in water supply could be partly explained by the timing and duration of the processes of trunk and leaf growth in the interaction with latex production. On the one hand, our data showed that rubber trees were characterized by a decoupling of the leaf and root phenology. Most of the leaves were produced within 2–4 weeks after the complete shedding of the trees in the middle of the dry season, when the SWC in the soil layer explored by the rhizotrons was at its lowest and the FR growth had almost ceased. Moreover, the dynamics of the PAI in our plantation showed that the duration of leaf growth was only 2 months while the duration of FR growth was about 10 months. However, on the other hand, Silpi et al. (2006) showed that the radial growth of the trunk was strongly reduced within 2 weeks from the beginning of the tapping season. Consequently, we could assume that most of the annual radial trunk growth occurred during the period when the trees were not being tapped, that is from the end of January to late April/early May. Therefore, most of the leaf and trunk growth was not exposed to the intermittent water stress events during the rainy season that greatly impacted on the FRP. Besides, a proper estimation of the contribution of the root system to the NPP should take into account deep roots (Maegth et al., 2013). Our data do not tell us anything about the behavior of the FRs below the maximum depth explored by the rhizotrons used in this study (75 cm). Meier and Leuschner (2008) found a shift with decreasing precipitation of the FR growth from the top soil to deeper layers in European beech stands. Under soil and climatic conditions similar to those in the current study, Gonkhamdee et al. (2009) showed that the growth of FRs below a soil depth of 75 cm in a 12-year-old rubber stand occurred mostly between July and November after the FRs had stopped growing in the upper layers. Thereby, the FR growth at deeper layers could have compensated for the water-stress-limited growth in the upper layers.

## CONCLUSION

The FR elongation rate and the FRP of field-grown rubber trees showed marked seasonal and inter-annual variations. The seasonal changes clearly relate to rainfall and soil water availability with the appearance of new roots and root growth being highly sensitive to slight decreases in the soil water potential during the rainy season. We also found that FR dynamics were also depressed by the tapping of the trunk for latex harvesting. This result demonstrates that tapping disturbed the carbon dynamics in the whole tree far beyond the area of the trunk where it was performed. In this regard, we recommend that greater attention be paid to the diversity of existing tapping systems in further studies on the carbon balance of rubber plantations.

## Conflict of Interest Statement

The authors declare that the research was conducted in the absence of any commercial or financial relationships that could be construed as a potential conflict of interest.
